# Reactivity Ratios for Organotin Copolymer Systems

**DOI:** 10.3390/molecules15042749

**Published:** 2010-04-15

**Authors:** Mohamed H. El-Newehy, Salem S. Al-Deyab, Ali Mohsen Ali Al-Hazmi

**Affiliations:** Department of Chemistry, College of Science, King Saud University, Riyadh 11451, P.O. Box 2455, Saudi Arabia; E-Mails: mnewehy@hotmail.com (M.H.E.); petrochem@ksu.edu.sa (A.M.A.)

**Keywords:** organotin monomers, bis(tri-*n*-butyltin) oxide, maleate, itaconate, styrene, methyl methacrylate, reactivity ratio

## Abstract

Di(tri-*n*-butyltin) itaconate (DTBTI) and monoethyl tributyltin fumarate (METBTF) were synthesized as organotin monomers. The organotin monomers were copolymerized with styrene (ST) and methyl methacrylate (MMA) via a free radical polymerization technique. The overall conversion was kept low (°15% wt/wt) for all studied samples and the copolymer composition was determined from tin analysis. The synthesized monomers and copolymers were characterized by elemental analysis, ^1^H- and ^13^C-NMR, and FTIR spectroscopy.

## 1. Introduction

Copolymers containing both hydrophilic and hydrophobic segments (amphiphilic polymers) are drawing considerable attention because of their possible use in biological systems. Various copolymer compositions can produce a very large number of different arrangements, affording materials with varying chemical and physical properties [1]. Moreover, knowledge of a copolymer’s composition is an important factor in the evaluation of its utility [2,3,4]. Controlling the polymer property parameters, such as copolymer composition, copolymer sequence distribution and molecular weight averages, is of particular importance in copolymerization processes [2]. To calculate the polymerization rate or polymer productivity and copolymer composition, monomer reactivity ratios must be known [5]. Reactivity ratios are among the most important parameters for the composition equation of copolymers, which can offer information such as the relative reactivity of monomer pairs and help estimate the copolymer composition [2,3]. Determination of the monomer reactivity ratios with small confidence intervals requires sensitive analytical techniques, careful planning of experiments and the use of statistically valid methods of estimation [5,6]. The method which is used most often nowadays for estimating monomer reactivity ratios is to perform low conversion copolymerization at various initial monomer feed compositions. Subsequently, the copolymer composition is determined for each reaction. Traditional methods for estimating monomer reactivity ratios are based on, first, transforming the instantaneous copolymer composition equation into a form that is linear in the parameters *r*_1_ and *r*_2_ and then estimating the monomer reactivity ratios by graphical plotting or by the linear least-squares method [7,8,9]. Linearization of the copolymer composition equation will distort the error distributions associated with the data.

Copolymers based on methacrylate monomers are an important base for oil additives with several functions, such as viscosity index improver, pour-point depressant and anti-foam agent [5]. Moreover, organotin compounds have important applications in several areas and hence they are made industrially on a large scale. The organotin moiety is attached to the monomers and copolymers via O-Sn and/or N-Sn bonds [2,10,11,12,13,14,15]. Acrylic copolymers with pendant organotin moieties find widespread applications as antifouling agents, wood preservatives, fungicides, pesticides, mosquito larvacides, heat and light stabilizers in the manufacture of poly(vinyl chloride) and biological activities against various species [2,16,17,18].

In our paper di(tri-*n*-butyltin) itaconate (DTBTI) and monoethyl tributyltin fumarate (METBTF) were synthesized as organotin monomers. The structural characterization of their copolymers with styrene (ST) and methyl methaacrylate (MMA) was perfomed and the reactivity ratios in the copolymerization determined for the classical copolymerization model using the Finemann–Ross linearization method (FR method) [2,19].

## 2. Results and Discussion

### 2.1. Synthesis of Organotin Monomers

**Scheme 1:**
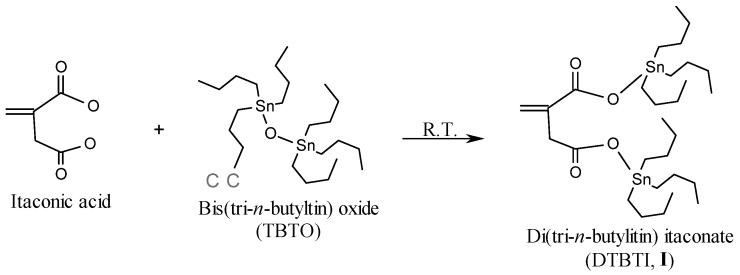
Synthesis of di(tri-*n*-butyltin) itaconate (DTBTI, **I**).

**Scheme 2:**
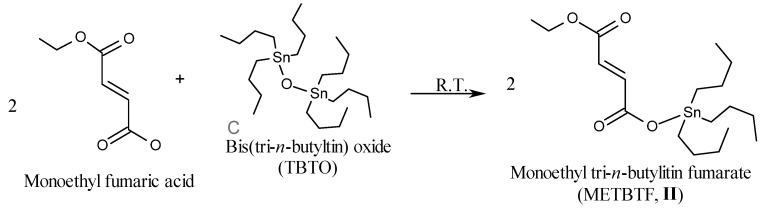
Synthesis of monoethyl tri-*n*-butyltin fumarate (METBTF, **II**).

The organotin monomers DTBTI (**I**) and METBTF (**II**), were prepared via esterfication of the carboxylic acid groups of itaconoic acid and monoethyl fumarate with bis(tri-n-butyltin) oxide (TBTO) at room temperature in 1:2 and 2:1 ratio, respectively (Scheme 1 and Scheme 2). The purity of the prepared monomers was checked by Thin Layer Chromatography (TLC) using ethyl acetate/cyclohexane (1:1) as eluent. The structures were elucidated by elemental analysis, FTIR, and ^1^H- and ^13^C-NMR. Generally, elemental microanalyses, as shown in Table 1, were in a good agreement with the calculated values.

**Table 1:** Elemental microanalyses of monomers **I** and **II**.

**Monomer**	Calc.			Found		
	%C	%H	%Sn	%C	%H	%Sn*
**I**	49.19	8.26	33.52	48.39	8.41	33.49
**II**	49.99	7.91	27.40	50.23	8.23	27.30

* Sn was estimated using the Gilman and Rosenberg method [4].

The FT-IR spectra of DTBTI (**I**) and METBTF **II**) showed characteristic peaks at: 2,852, 2,922 and 2,954 cm^-1^ assigned to C-H stretching (-C*H_2_*C*H_2_*CH_2_CH_3_, -CH_2_=C-, and -CH=CH-), 1,698, 1,715 and 1,728 cm^-1^ assigned to the overlap of the carbonyl groups (C=O stretching) and 1,630 cm^-1^ assigned to C=C stretching. The esterfication of the carboxylic group was confirmed by the disappearance of the hydroxyl group broad bands of itaconic acid and fumaric acid at 2,800–3,200 cm^-1^.

The ^1^H-NMR spectrum of DTBTI showed peaks (δ, ppm) at: 0.84–0.86 (triplet, CH_2_CH_2_CH_2_C*H_3_*), 1.20–1.30 (multiplet, -CH_2_CH_2_C*H_2_*CH_3_), 1.56 (multiplet, CH_2_C*H_2_*CH_2_CH_3_), 3.27 (multiplet, C*H_2_*CH_2_CH_2_CH_3_), 5.52 and 6.22 (singlet, C*H_2_*=C-), 6.68 and 6.82 (doublet, -C*H*=C*H*-). The ^1^H-NMR spectrum of METBTF showed peaks (δ, ppm) at: 0.84 (triplet, -CH_2_CH_2_CH_2_C*H_3_*), 1.21–1.28 (multiplet, -CH_2_CH_2_C*H_2_*CH_3_), 1.55 (triplet, -CH_2_C*H_2_*CH_2_CH_3_), 4.17 (quartet, COOCH_2_CH_3_), 6.68 and 6.82 (doublet, -C*H*=C*H*-). Generally, the esterification of carboxylic groups was confirmed by the disappearance of the acidic proton peak at δ 12.80 ppm due to the replacement by tin.

The ^13^C-NMR spectrum of **I** showed peaks (δ, ppm) at: 13.71, 16.46, 27.12, 27.77 (*C*H_2_*C*H_2_*C*H_2_*C*H_3_), 38.91 (-C-*C*H_2_-COO-), 126.07 (*C*H_2_=C-), 136.99 (CH_2_=*C*-), 171.61 (=C-*C*OO-), 176.61 (-CH_2_-*C*OO-), while that of **II** showed peaks (δ, ppm) at: 13.61, 16.65, 27.66, 27.76 (-*C*H_2_*C*H_2_*C*H_2_*C*H_3_) and (-COOCH_2_C*C*H_3_), 60.97 (-CH=CH-COO*C*H_2_CH_3_), 132.30 ( *C*H=*C*H-COOSnBu_3_), 136.05 (-CH=*C*H-COO*C*H_2_CH_3_), 165.63 (-CH=CH-*C*OO*C*H_2_CH_3_), 169.74 (-CH=CH-*C*OOSnBu_3_).

### 2.2. Copolymer Synthesis

**Scheme 3:**
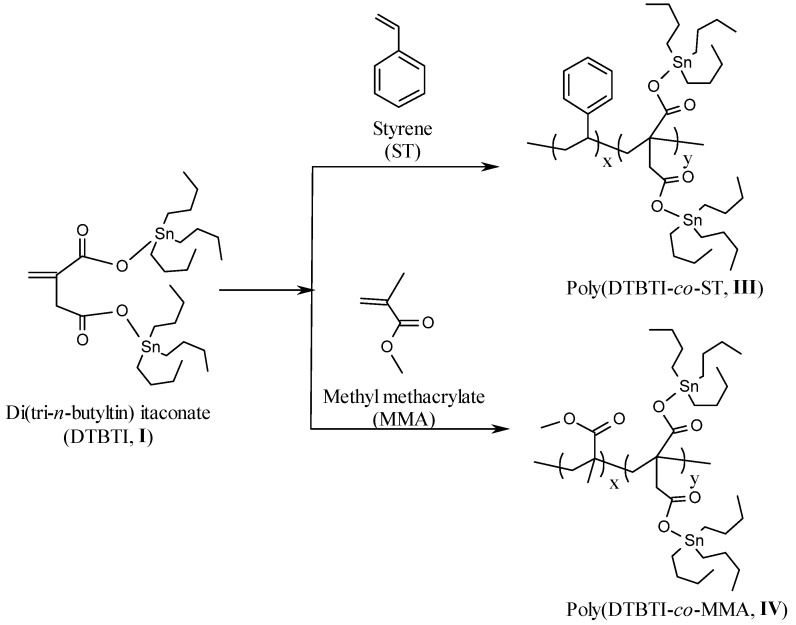
Copolymerization of di-(tri-*n*-butyltin) itaconate (DTBTI) with styrene (ST) and methyl methacrylate (MMA).

**Scheme 4:**
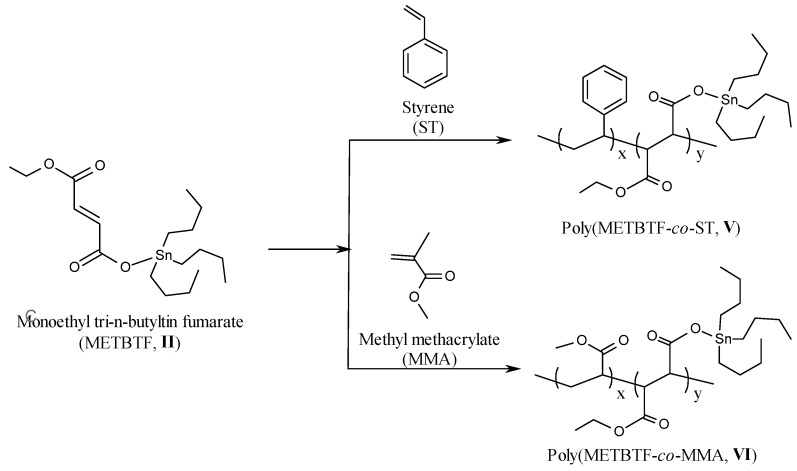
Copolymerization of monoethyl tri-*n*-butyl fumarate (METBTF) with styrene (ST) and methyl methacrylate (MMA).

Copolymerization of DTBTI (**I**) or METBTF (**II**) with styrene (ST) or methyl methacrylate (MMA) was done via a free radical polymerization technique with a total concentration of 2 mol/L for different time intervals (Scheme 3 and Scheme 4). Different copolymers with different ratios were thus prepared and the percentage of tin was determined in each sample (Table 2) [20].

**Table 2:** The experimental tin percentages of copolymers **III–VI** with different ratios.

Copolymer Ratio	%Sn			
	III ^a^	IV ^b^	V ^c^	VI ^d^
10/90	21.54	10.66	10.92	2.24
20/80	20.03	9.16	11.66	2.12
30/70	18.28	7.17	11.34	1.51
40/60	15.54	5.18	8.05	1.17
50/50	10.73	3.95	5.50	0.65

^a^ DTBTI/ST; ^b^ DTBTI/MMA; ^c^ METBTF/ST; ^d^ METBTF/MMA.

### 2.3. Structural Characterization of the Copolymers ***III-VI***

The structural characterizations of copolymers **III-VI** were done by FTIR and ^1^H-NMR spectroscopy. The FT-IR spectra of **III**, **IV**, **V** and **VI** with overall conversions of 7.81% (3 h), 9.92% (2 h), 8.59% (3 h) and 7.24% (0.5 h), respectively, was characterized by the disappearance of the C=C stretching band of DTBTI, METBTF, ST, and BA at 1,630 and 1,640 cm^-1^ , respectively, which confirm the formation of the copolymer. Generally, the FT-IR spectra showed peaks at 1,452, 1,492, 1,617 and 1,637 cm^-1^ assigned to C=C stretching of the styrene aromatic ring. The FTIR spectra showed characteristic peaks at 1,605 and 1,736 cm^-1^ assigned to C=O stretching, in addition to peaks at 2,850, 2,924, 3,025, 3,058, and 3,080 cm^-1^ assigned to C-H stretching of the aromatic ring. 

The ^1^H-NMR spectra of **III**, **IV**, **V** and **VI** were characterized by the disappearance of peaks at δ 5.51 and 5.49–6.04 ppm (-C*H*=C-, -C*H*=CCH_3_- and C*H_2_*=C*H*-) of DTBTI, METBTF, ST and MMA, respectively, which confirm the formation of the copolymers. The ^1^H-NMR spectrum of **III** was characterized by peaks at δ 0.80–1.53 ppm (‑C*H_2_*C*H_2_*C*H_2_*C*H_3_*, -C*H_2_*-C*H*Ph-, and -C*H*-C*H*-), and at δ 6.50–7.00 ppm (*H_arom_*). The ^1^H-NMR spectrum of **IV** was characterized by the presence of peaks at δ 0.80–1.60 ppm C*H_2_*C*H_2_*C*H_2_*C*H_3_*, ‑COOC*H_3_*, -C*H*-CHCOO-) and at δ 3.57 ppm (-C*H*-C*H*-, -CH-C*H*COO-). The ^1^H-NMR spectrum of **V** was characterized by peaks at δ 0.80–1.41 ppm (‑C*H_2_*C*H_2_*C*H_2_*C*H_3_*, -C*H_2_*-C-COO-, and -CH-CHC*H_3_*-), and at δ 6.50–7.00 ppm (*H_arom_*). The ^1^H-NMR spectrum of **VI** was characterized by peaks at δ 0.80–1.39 ppm (-C*H_2_*C*H_2_*C*H_2_*C*H_3_*, ‑COOC*H_3_*, -C*H*-CHCOO-) and at δ 3.56 ppm (-C*H*-C*H*CH_3_-, -CH-C*H*COO-)*.*

### 2.4. Reactivity Ratio Determination

Copolymers **III-VI** were prepared using different ratios of the corresponding monomers using BPO as initiator and the polymerization was stopped at overall conversions ≤15wt/wt%. Copolymers **III, V** and **VI** were precipitated in methanol and copolymer **IV** was precipitated in petroleum ether (b.p. 60–80 °C). The percentage of tin was calculated according to Gilman and Rosenberg method [19], and subsequently the copolymer composition (f) of copolymers **III-VI** was determined as shown in Table 3.

The monomer reactivity ratios and the content of the reaction mixture and the copolymer were calculated according to the R method [19,21,22] (Table 4). Generally, from the values of the experimental reactivity ratios, tt is evident that r_1_ (*k_11_/k_12_*) is negative so it will correct to zero [19,23,24,25]. As r_1_ = 0, DTBTI and METBTF cannot form homopolymers (*i.e., k_11_*= 0). As r_1_r_2_ = 0, so the copolymer trend will tend to form a homopolymer from the active monomer, *i.e.,* the copolymerization will prefer the formation of MMA or ST homopolymer.

**Table 3:** The composition parameters of copolymers **III–VI**.

Copolymer		% Sn	M_1_^a^	F ^b^	m_1_^c^	F ^d^	Conversion (wt/wt%) ^f^
**Code**	**Ratio**
III	10/90	10.73	7.81	0.0689	0.0645	0.1111	0.1
	20/80	15.54	9.07	0.1264	0.1122	0.25	0.2
	30/70	18.28	8.33	0.1754	0.1492	0.428	0.3
	40/60	20.03	6.62	0.2169	0.1782	0.667	0.4
	50/50	21.55	5.32	0.2626	0.20795	1.0	0.5
IV	10/90	60	9.92	0.0257	0.0257	0.1111	0.11
	20/80	85	7.27	0.0383	0.0383	0.1764	0.15
	30/70	108	5.94	0.0529	0.0530	0.25	0.20
	40/60	128	5.27	0.0656	0.0656	0.3333	0.25
V	10/90	5.50	8.59	0.0587	0.0554	0.1111	0.1
	20/80	8.05	6.39	0.0945	0.0864	0.250	0.2
	30/70	11.34	3.39	0.1528	0.1326	0.428	0.3
	40/60	11.66	1.42	0.1595	0.1375	0.667	0.4
	50/50	10.92	0.43	0.1444	0.1262	1.000	0.5
VI	10/90	0.65	7.24	0.0056	0.055	0.1111	0.1
	20/80	1.17	6.26	0.0102	0.0101	0.25	0.2
	30/70	1.51	7.84	0.0134	0.0132	0.428	0.3
	40/60	2.12	5.65	0.0193	0.0189	0.667	0.4
	50/50	2.24	3.96	0.0204	0.0200	1.000	0.5

^a^ Mole fraction of DTBTI or METBTF in reaction mixture; ^b^ Molar ratio of DTBTI or METBTF to ST or MMA in reaction mixture; ^c^ Mole fraction of DTBTI or METBTF in copolymer; ^d^ Molar ratio of DTBTI or METBTF to ST or MMA in copolymer; ^f^ Overall conversion.

**Table 4:** Monomer reactivity ratios and the FR parameters of copolymers **III–VI**.

Copolymer		Monomer Ratio F = M_1_/M_2_	M-Unit Ratio in Copolymer	Parameters of FR Eq.	
Code	Ratio			F^2^ /f	F/f(f-1)
III	10/90	0.1111	0.0645	0.1787	-1.4987
	20/80	0.25	0.1122	0.4943	-1.7273
	30/70	0.428	0.1492	1.0446	-2.0127
	40/60	0.667	0.1782	2.0516	-2.4088
	50/50	1.0	0.2079	3.8088	-2.8088
IV	10/90	0.1111	0.0257	0.4789	-4.2031
	20/80	0.1674	0.0383	0.8124	-4.429
	30/70	0.25	0.0529	1.1816	-4.4765
	40/60	0.3333	0.0656	1.6932	-4.7468
	50/50	0.1111	0.0257	0.4789	-4.2031
V	10/90	0.1111	0.0587	0.2103	-1.7821
	20/80	0.25	0.0945	0.6612	-2.3946
	30/70	0.428	0.1528	1.1988	-2.3729
	40/60	0.667	0.1595	2.7901	-3.5161
	50/50	1.0	0.1444	6.9269	-5.9269
VI	10/90	0.111	0.0056	2.1960	-19.654
	20/80	0.250	0.0103	6.0860	-24.094
	30/70	0.428	0.0134	13.6642	-31.498
	40/60	0.667	0.0193	23.0147	-33.838
	50/50	1.000	0.0209	48.8220	-47.822

## 3. Experimental

### 3.1. Materials

Itaconic acid 99% was purchased from Sigma-Aldrich. Bis(tri-*n*-butyltin) oxide, monoethyl fumarate and styrene (ST) were purchased from Fluka. Methyl methacrylate and benzoyl peroxide (BPO) were purchased from BDH. 2,2^'^ -Azobisisobutyronitrile (AIBN) was purchased from Riedel-de- Haen. All solvents were purchased from BDH and used as received.

### 3.2. Characterization

^1^H- and ^13^C-NMR Spectra were recorded on a Jeol (400 MHz) instrument. FTIR spectra were recorded on a Perkin Elmer 883. Elemental analyses were performed on a Perkin Elmer Series II CHN/O Analyzer 2400. Thin-layer chromatography (TLC) was performed using the ascending technique with precoated silica gel 60F 254 on aluminum sheets.

### 3.3. Synthesis of Organotin Monomers

#### 3.3.1. Synthesis of Di(tri-*n*-butyltin) Itaconate (DTBTI, **I**)

This monomer was prepared according to the method of Cummins and Dunn [26], which may be summarized as follows: bis(tri-*n*-butyltin) oxide (29.8 g, 50.0 mmol) was added dropwise at room temperature within 2 hrs to itaconic acid (6.5 g, 50.0 mmol) contained in a 500 mL round bottom flask,. The reaction mixture was stirred at room temperature for 8 h, and then heated at 110 °C for 1.5 h. The mixture was solidified by heating in a vacuum oven at 45 °C for 2 h. The product I was recrystallized from petroleum ether (40–60 °C) and was dried under vacuum at 40 °C for 24 h to give 28.0 g (79.0% yield) of the title compound, m.p. 54–56 °C.

#### 3.3.2. Synthesis of Monoethyl Tri-*n*-butyltin Fumarate (METBTF, **II**)

The title monomer was prepared as described earlier for DTBTI using the following quantities: monoethyl fumarate (7.2 g, 25.0 mmol), bis(tri-*n*-butyltin) oxide (14.9 g, 50.0 mmol). The product **II** was recrystallized from petroleum ether (b.p. 40–60 °C) and dried under vacuum at 40 °C for 24 h to give 11.5 g (53.1% yield) of **II**, m.p. 42–44 °C.

### 3.4. General Procedure for Copolymerization

Copolymerizations were carried out in a three necked round bottomed flask by dissolving benzoyl peroxide (BPO, 1% mol) in 2 mL of the corresponding solvent, and then the calculated molar quantities of the monomers were added. The polymerization mixtures were bubbled with nitrogen to expel oxygen. Copolymerization was done at 70 °C for the desired period of time. The formed copolymer was precipitated in an excess amount (20 fold), of the corresponding solvent. All samples were dried in an oven under vacuum at 40–60 °C. For reactivity ratio determination, the copolymerization was stopped at overall conversion below 15% wt/wt [2,27] from the total weight of both monomers by changing the time of polymerization [2,28].

### 3.5. Reactivity Ratios Determination

For reactivity ratio determination, copolymerizations were performed with different initial feed ratios while maintaining the monomer conversion below 10%. The Fineman–Ross (FR) method was employed. The initiator concentration was kept at 1% relative to the total monomers concentration in benzene or DMF. Monomer reactivity ratios can be calculated from the experimental results depending on the copolymer composition. Copolymer composition can be expressed as follows:

f_1_ = m_1_/m_2_ and f_2_ = m_2_/m_1_

Where m_1_ and m_2_ are the mole fractions of DTBTI or METBTF and vinyl monomer in the copolymer, respectively, and f_1_ and f_2_ are its molar ratios in the copolymer. Moreover, the feed composition of the reaction mixture is known in advance, so feed composition was used in the calculations of the reactivity ratios and can be expressed as follows:

F_1_ = M_1_/M_2_ and F_2_ = M_2_/M_1_

where M_1_ and M_2_ are the mole fractions of DTBTI or METBTF and vinyl monomer in the reaction mixture, respectively, and F_1_ and F_2_ are its Molar ratios in the feed composition.

In this research, the calculations were based on the tin content in the copolymer composition [29,30]. The Fineman–Ross (FR) [19] method was based on the use of copolymer composition and the content of the polymerization mixture. Copolymer composition and feed composition were calculated according to equation (2):

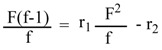
(2)

A plot of (F^2^ /f) on X-axis *vs.* {F/f (f-1)} on Y-axis gave a straight line, the intercept is r_2_ and the slope is r_1_.

## 4. Conclusions

Organotin monomers, di(tri-*n*-butyltin) itaconate (DTBTI) and monoethyl tri-*n*-butyltin maleate (METBTF), were synthesized. The organotin monomers were copolymerized with styrene (ST) and methyl methacrylate (MMA) using a free radical technique. The overall conversions were kept low (≤15% wt/wt) for all studied samples and the copolymer compositions were determined by tin analysis. From the values of the experimental reactivity ratio, r_1_ = 0, so DTBTI and METBTF cannot form homopolymer (*i.e. , k_11_* = 0). Moreover, as r_1_r_2_ = 0, so the copolymer trend will tend to form a homopolymer from the active monomer, *i.e.,* the copolymerization is preferred to the formation of ST and MMA homopolymer.

*Sample Availability:* Samples of the compounds are available from the authors.
